# Pregnancy outcomes in women affected by fetal alpha-thalassemia: a case control study

**DOI:** 10.1038/s41598-021-95998-1

**Published:** 2021-08-27

**Authors:** Jiangheng Li, Jingli Yan, Yongquan Huang, Jinlu Wei, Bingyan Xie, Maoling Zhu, Wu Jiang

**Affiliations:** Department of Maternity-Child Health and Family Planning Services, Affiliated Nanning Maternal and Child Health Hospital of Guangxi Medical University, No. 20 Liwan Road, Qingxiu District, Nanning City, 530022 Guangxi Province China

**Keywords:** Diseases, Risk factors

## Abstract

To evaluate the possible associations between fetal α-thalassemia and risk of adverse pregnancy outcomes using a provincial woman-child health service information database in China. This was a case control study (N = 438,747) in which we compared all singleton pregnancies of women with or without the α-thalassemia trait from May 2016 to May 2020, and where women with the trait were further allocated to a normal fetal group, a group of fetuses with the α-thalassemia trait, and a fetal group with hemoglobin H (HbH) disease according to the results of fetal DNA analysis. With thalassemic women whose fetuses were normal as the reference, fetuses in the HbH disease group showed a higher increase in the odds of Apgar scores being < 7 at 1 min (adjusted odds ratio [aOR], 2.79; 1.03–7.59) and 5 min (aOR, 4.56; 1.07–19.40). With non-thalassemic women as the reference, these trends were more obvious (aOR, 4.83; 2.55–9.16; aOR, 6.24; 2.75–14.18, respectively); whereas the normal fetal group was more likely to be diagnosed with postpartum hemorrhage (aOR, 1.66; 1.10–2.50). In addition, fetal HbH disease and gestational age were two independent factors influencing low Apgar scores, and their combination reflected medium accuracy in Apgar predictions.

## Introduction

Alpha (α)-thalassemia is an autosomal recessive disease that is caused by deletion or mutation of one or both of the linked α-globin genes that are present on the short arm of each chromosome 16 (16p13.3)^1–3^. α-Thalassemia is comprised of α^+^-thalassemia and α^0^-thalassemia. α^+^-Thalassemia occurs as a result of deletions (e.g., –α^3.7^ and –α^4.2^) or point mutations (e.g., α^CS^α and α^QS^α) in the single α-globin gene; whereas α^0^-thalassemia, the more severe form of α-thalassemia, is usually caused by deletions of larger fragments that involve both linked α-globin genes (e.g., –^SEA^ and –^THAI^)^4^.

The α^+^-thalassemia mutation—which occurs at a frequency of 10–25% in tropical areas such as sub-Saharan Africa, Mediterranean regions, the Middle East, South Asia, and Southeast Asia—is the most common monogenic disease observed worldwide^5,6^. Compared to α^+^-thalassemia, α^0^-thalassemia is much less common, but spread at a high frequency in the Mediterranean basin and particularly in Southeast Asia, including Southern China^7^. Guangxi province is located in the southern regions of China, in which the prevalence of α-thalassemia is over 14%, whereas the overall prevalence in China is 7.88%, and this undoubtedly represents the highest prevalence of any region in China^8–10^. In addition, the –^SEA^ variant is found to be the most common mutation in mainland China, accounting for approximately 50% of α-thalassemia in Guangxi province^8,10,11^. α-Thalassemia has thus become a major public health issue in China, especially in Guangxi province.

The α-thalassemia trait, one form of mild microcytic hypochromic anemia, is usually observed in α^+^-thalassemia homozygotes or α^0^-thalassemia heterozygotes^4^. Hemoglobin H (HbH) disease (the compound heterozygous condition for α^+^-thalassemia and α^0^-thalassemia) can present notable phenotypic variability—varying from asymptomatic, to severe hemolytic anemia, and even to hydrops fetalis syndrome in utero—and it is particularly prevalent in southern China^12^. Some investigators have recently suggested that women with the α-thalassemia trait or HbH disease exhibit lower hemoglobin (Hb) levels that are significantly associated with increased risk for adverse pregnancy outcomes such as preterm birth and low birth weight, when compared with women without thalassemia^13–15^. However, there are few studies on the influences of fetuses suffering from these disease on outcomes of pregnancy.

Our objective was to investigate the possible associations between fetuses with the α-thalassemia trait or HbH disease and adverse pregnancy outcomes among women by using a population-based case-control study. We posited that this study would provide more comprehensive and accurate risk estimates for both parents with α-thalassemia so as to reduce the incidence of adverse pregnancy outcomes.

## Methods

### Study population

In Guangxi province, information from antenatal, delivery, newborn, and child death records for all births in which newborns delivered after 20 weeks of gestation is abstracted from the medical records into the Guangxi Woman and Child Health Service Information System, a provincial database administered by the Guangxi Health Commission. All information is then inputted by health information management professionals who undergo a training program every year. Data quality is maintained by ongoing hospital self-checks, municipal or provincial-level quality checks, and checks in the data-entry software program.

**Figure 1:**
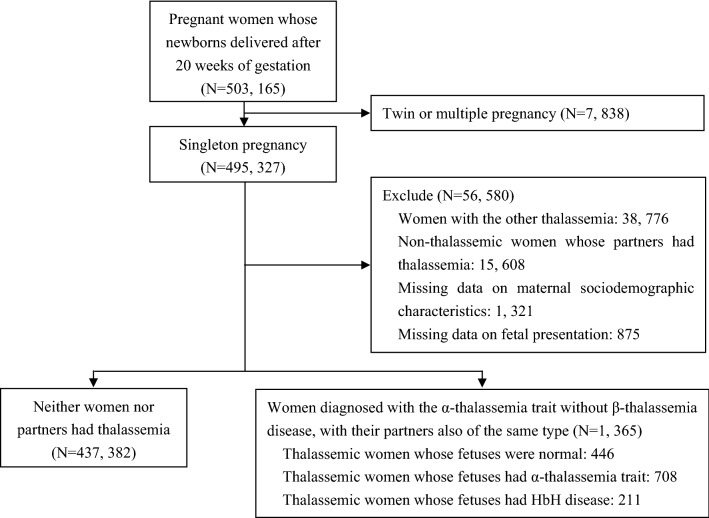
Study flow diagram.

We conducted a population-based case-control study in which we compared pregnancy outcomes of women with and without the α-thalassemia trait, and further allocated women with the trait to a normal fetal group, a group with fetuses manifesting the α-thalassemia trait, and a group with fetuses exhibiting HbH disease. Pregnant women and their partners were first screened using the red blood cell indices [mean cell volume (MCV) and mean cell hemoglobin (MCH)] and hemoglobin electrophoresis. If MCV was < 82 fl, MCH was < 27 pg, HbA2 concentration was > 3.5% or < 2.5%, HbF was increased, or abnormal hemoglobin bands occurred, he or she would be considered a possible thalassemia carrier and be further characterized by molecular diagnosis as described previously^11^. Next, couples with same type of thalassemia were recommended to undergo a prenatal diagnostic procedure as described previously^4^ that would identify thalassemic genotypes of their fetuses. We recruited all pregnant women who delivered at Nanning, the capital of Guangxi province, between May 2016 and May 2020. The inclusion criteria for the study group were as follows: (1) pregnant women diagnosed with the α-thalassemia trait without β-thalassemia disease, with their partners also of the same type; (2) thalassemic women whose fetuses had non-thalassemia, α-thalassemia trait or HbH disease as identified by invasive prenatal diagnosis; (3) singleton pregnancy; and (4) the presence of available data for clinical characteristics and pregnancy outcomes. Pregnant women who delivered at Nanning in the same period were recruited as the control group. The inclusion criteria for the control group were as follows: (1) neither mother nor partner had thalassemia; and (2) singleton pregnancy with available data of clinical characteristics and pregnancy outcome. The flow diagram for study participants was shown in Fig. 1. Ethics approval was obtained from the Ethics Committee of Affiliated Nanning Maternal and Child Health Hospital of Guangxi Medical University, and all participants or their legal guardians provided written informed consent. All methods were carried out in accordance with relevant guidelines and regulations^4,11,13^. Maternal clinical characteristics, pregnancy outcomes—as well as data from maternal and fetal genotypes in our study—came from the Guangxi Woman and Child Health Service Information System.

### Definition and outcomes

The following clinical characteristics were evaluated: maternal age, maternal anemia during pregnancy (hemoglobin less than 10 g/dL), number of prenatal visits greater than 10, gestational weeks at first visit, gravidity, parity, ethnicity, and residency in Guangxi province. We also assessed the following pregnancy outcomes: cesarean delivery, postpartum hemorrhage (the loss of more than 500 mL of blood within the first 24 h following childbirth), birth before 34 weeks of gestation, birth before 37 weeks of gestation, fetal growth restriction (birth weight less than the 10th percentile of the normal growth curve), low birth weight (birth weight less than 2500 g), macrosomia (birth weight not less than 4000 g), Apgar scores at 1 and 5 min less than 7, and perinatal death (death in utero after 20 weeks of gestation or within 7 days of birth).

### Statistical analysis

We analyzed differences among all four groups or three subgroups according to demographic variables and prevalence of adverse pregnancy outcomes using χ^2^ or the Fisher–Freeman–Halton exact test. To uncover the potential associations between fetal α-thalassemia and pregnancy outcomes, binary logistic regression analyses were performed where we compared each of the study groups with their reference groups. In addition, we constructed a binary logistic regression model with backward elimination (Backward Likelihood Ratio) to explore any independent factors influencing low Apgar scores. The above statistical analyses were performed using SPSS version 17.0 (SPSS, Chicago, IL, USA). We used receiver operating characteristic (ROC) curve analysis, as calculated with MedCalc Statistical Software version 15.2.2 (MedCalc Software Bvba, Ostend, Belgium), to evaluate the ability of the various factors in predicting low Apgar scores. The data points for the ROC curve were derived exclusively from subjects with the α-thalassemia trait. The descriptive data are presented as numbers (percentages). The measure of association between exposure and outcome was expressed as odds ratios (ORs) with 95% confidence intervals (CIs). A value of *P* < 0.05 was considered statistically significant.

## Results

### Clinical characteristics of subjects

Nearly all of pregnant women were Chinese nationals residing in Guangxi province. As depicted in Table 1, there were no statistical differences in any baseline characteristic of thalassemic women among the three subgroups. Four groups were comparable in terms of maternal age, gestational weeks at first visit and gravidity, but maternal anemia, parity, ethnic group, residency in Guangxi province, and the number of prenatal visits were statistically different. The rate of maternal anemia was significantly higher in the normal fetal group, fetal group with the α-thalassemia trait, and the fetal group with HbH disease compared with the control group. In addition, we noted higher percentages of nulliparity, Zhuang ethnicity, residency in Guangxi province, and the number of prenatal visits > 10 among thalassemic women relative to controls.

**Table 1:** Baseline characteristics of thalassemic women and controls.

Characteristics	Control group (n = 437,382)	Women with the α-thalassemia trait*			*P*_1_^§^ (*P*_2_^║^)
		Normal fetal group (n = 446)	Fetal group with α-thalassemia trait^†^ (n = 708)	Fetal group with HbH disease^‡^ (n = 211)	
**Maternal age (y)**					0.100 (.254)
12–19	12,956 (2.96)	11 (2.47)	18 (2.54)	11 (5.21)	
20–34	346,994 (79.33)	353 (79.15)	573 (80.93)	155 (73.46)	
35–58	77,432 (17.70)	82 (18.39)	117 (16.53)	45 (21.33)	
Maternal anemia (Hb < 10 g/dL)	37,864 (8.66)	68 (15.25)	133 (18.79)	42 (19.91)	0.213 (< .001)
Number of prenatal visits > 10	206,526 (47.22)	245 (54.93)	376 (53.11)	118 (55.92)	0.709 (< .001)
**Gestational weeks at first visit**					0.051 (.062)
< 13	320,425 (73.26)	317 (71.08)	526 (74.29)	139 (65.88)	
13–42	116,957 (26.74)	129 (28.92)	182 (25.71)	72 (34.12)	
**Gravidity**					0.461 (.069)
1	100,526 (22.98)	105 (23.54)	188 (26.55)	57 (27.01)	
2–23	336,856 (77.02)	341 (76.46)	520 (73.45)	154 (72.99)	
**Parity**					0.080 (< .001)
Nulliparous	165,076 (37.74)	213 (47.76)	331 (46.75)	117 (55.45)	
Parous	272,306 (62.26)	233 (52.24)	377 (53.25)	94 (44.55)	
**Ethnic group**					0.311 (< .001)
Han	228,462 (52.23)	185 (41.48)	323 (45.62)	94 (44.55)	
Zhuang	193,659 (44.28)	244 (54.71)	371 (52.40)	111 (52.61)	
Others	15,261 (3.49)	17 (3.81)	14 (1.98)	6 (2.84)	
Residency in Guangxi province	411,730 (94.14)	442 (99.10)	702 (99.15)	209 (99.05)	0.989 (< .001)

Data are n (%) unless otherwise specified. *Includes two types of heterozygous mutations, –^SEA^/αα (99.12%) and –^THAI^/αα (0.88%). ^†^Includes two types of heterozygous mutations, –^SEA^/αα (98.45%) and –^THAI^/αα (1.55%). ^‡^There were 184 deletional HbH diseases (–/–α) and 27 non-deletional HbH diseases (–/α^T^α). Deletional HbH diseases comprised –^SEA^/–α^3.7^ (64.67%) and –^SEA^/–α^4.2^ (35.33%), while non-deletional HbH diseases comprised –^SEA^/α^CS^α (92.59%) and –^SEA^/α^QS^α (7.41%). ^§^Chi-squared test for three subgroups. ^║^Chi-squared test for all groups.

### Maternal and neonatal outcomes

As shown in Table 2, a larger proportion of fetuses with HbH disease were likely to show low Apgar scores compared with the normal fetal group, but cesarean delivery, postpartum hemorrhage, birth before 34 or 37 weeks of gestation, fetal growth restriction, low birth weight, macrosomia, and perinatal death were not statistically different among the three subgroups. Compared with the control group, the proportion with low Apgar scores was also significantly larger among fetuses with HbH disease. Interestingly, the rate of postpartum hemorrhage was mildly higher in the normal fetal group compared with the controls. Although there was no statistical difference in the rate of low birth weight among the four groups, there was a statistical difference between the control group and the group of fetuses with HbH disease (*P* = 0.012). The differences in the proportions with respect to cesarean delivery, birth before 34 or 37 weeks of gestation, fetal growth restriction, macrosomia, and perinatal death were not statistically different among the four groups.

**Table 2:** Comparison of pregnancy outcomes among the four groups.

Outcome	Control group (n = 437,382)	Women with α-thalassemia trait			*P*_1_* (*P*_2_^†^)
		Normal fetal group (n = 446)	Fetal group with α-thalassemia trait (n = 708)	Fetal group with HbH disease (n = 211)	
Cesarean delivery	127,851 (29.23)	137 (30.72)	208 (29.38)	63 (29.86)	0.889 (.914)
Postpartum hemorrhage	14,232 (3.25)	24 (5.38)	30 (4.24)	9 (4.27)	0.643 (.026)
Birth before 34 weeks of gestation	5221 (1.19)	8 (1.79)	6 (0.85)	4 (1.90)	0.283 (.398)
Birth before 37 weeks of gestation	25,205 (5.76)	25 (5.61)	48 (6.78)	17 (8.06)	0.477 (.333)
Fetal growth restriction	9198 (2.10)	7 (1.57)	15 (2.12)	6 (2.84)	0.551 (.758)
Low birth weight	22,695 (5.19)	23 (5.16)	40 (5.65)	19 (9.00)	0.129 (.088)
Macrosomia	12,986 (2.97)	13 (2.91)	27 (3.81)	6 (2.84)	0.640 (.622)
Apgar score < 7 at 1 min	4034 (0.92)	7 (1.57)	9 (1.27)	10 (4.74)	0.004 (< .001)
Apgar score < 7 at 5 min	1918 (0.44)	3 (0.67)	1 (0.14)	6 (2.84)	0.001 (.001)
Perinatal death	1874 (0.43)	1 (0.22)	1 (0.14)	2 (0.95)	0.147 (.359)

Data are n (%) unless otherwise specified. *Chi-squared or Fisher–Freeman–Halton exact test for three subgroups. ^†^Chi-squared or Fisher–Freeman–Halton exact test for all groups.

### Fetal α-thalassemia was associated with adverse pregnancy outcomes

All variables with regard to pregnancy outcome that were found to possess *P* values < 0.05 for all four groups using the Chi-squared test were entered into the logistic models. As depicted in Table 3, the associations did not change markedly after adjusting for maternal age, gravidity, parity, ethnic group, gestational weeks at first visit, number of prenatal visits, residency in Guangxi province, or fetal sex in the binary regression analyses compared with crude regression analyses. The final binary logistic regression models demonstrated that compared with the normal fetal group, fetuses with HbH disease showed a 179% increase in the odds of an Apgar score < 7 at 1 min (4.74% vs 1.57%; adjusted odds ratio [aOR], 2.79; 1.03–7.59), and a 356% increase in the odds of an Apgar score < 7 at 5 min (2.84% vs 0.67%; aOR, 4.56; 1.07–19.40), whereas fetuses with the α-thalassemia trait did not change. These results suggested that fetuses with HbH disease instead of the α-thalassemia trait were likely to have an increased risk for low Apgar scores.

**Table 3:** Unconditional binary logistic regression analysis of pregnancy outcomes with thalassemic women whose fetuses were normal as the reference group.

Outcome	Unadjusted OR	95% CI	*P* value	aOR*	95% CI	*P*
**Postpartum hemorrhage**						
Fetal group with α-thalassemia trait	0.78	0.45–1.35	0.371	0.80	0.46–1.39	0.426
Fetal group with HbH disease	0.78	0.36–1.72	0.542	0.76	0.34–1.68	0.496
**Apgar score < 7 at 1 min**						
Fetal group with α-thalassemia trait	0.81	0.30–2.18	0.674	0.79	0.29–2.17	0.649
Fetal group with HbH disease	3.12	1.17–8.32	0.023	2.79	1.03–7.59	0.044
**Apgar score < 7 at 5 min**						
Fetal group with α-thalassemia trait	0.21	0.02–2.01	0.176	0.20	0.02–1.95	0.262
Fetal group with HbH disease	4.32	1.07–17.45	0.040	4.56	1.07–19.40	0.040

*Adjusted for maternal age, gravidity, parity, ethnic group, gestational weeks at first visit, number of prenatal visits, residency in Guangxi province or fetal sex.

With the control group as the reference, fetuses with HbH disease were at an obviously higher risk of an Apgar score being < 7 at 1 min (4.74% vs 0.92%; aOR, 4.83; 2.55–9.16) and an Apgar score < 7 at 5 min (2.84% vs 0.44%; aOR, 6.24; 2.75–14.18), as shown in Table 4. Notably, the odds of postpartum hemorrhage were slightly increased in the normal fetal group (aOR, 1.66; 1.10–2.50) compared with the control group.

**Table 4:** Unconditional binary logistic regression analysis of pregnancy outcomes with women without thalassemia as the reference group.

Outcome	Unadjusted OR	95% CI	*P* value	aOR*	95% CI	*P*
**Postpartum hemorrhage**						
Normal fetal group	1.69	1.12–2.55	0.012	1.66	1.10–2.50	0.016
Fetal group with α-thalassemia trait	1.32	0.91–1.90	0.142	1.32	0.91–1.90	0.142
Fetal group with HbH disease	1.32	0.68–2.58	0.409	1.29	0.66–2.52	0.456
**Apgar score < 7 at 1 min**						
Normal fetal group	1.71	0.81–3.62	0.158	1.70	0.81–3.61	0.163
Fetal group with α-thalassemia trait	1.38	0.72–2.67	0.334	1.36	0.70–2.62	0.364
Fetal group with HbH disease	5.34	2.83–10.09	< 0.001	4.83	2.55–9.16	< 0.001
**Apgar score < 7 at 5 min**						
Normal fetal group	1.54	0.50–4.81	0.453	1.59	0.51–4.96	0.425
Fetal group with α-thalassemia trait	0.32	0.05–2.29	0.258	0.33	0.05–2.32	0.262
Fetal group with HbH disease	6.65	2.95–14.98	< 0.001	6.24	2.75–14.18	< 0.001

*Adjusted for maternal age, gravidity, parity, ethnic group, gestational weeks at first visit, number of prenatal visits, residency in Guangxi province or fetal sex.

### Low Apgar scores and associated factors

To identify the potential factors influencing low Apgar scores, we used binary logistic regression with backward elimination (Backward Likelihood Ratio) to analyze the thalassemic women, where only fetal α-thalassemia and gestational age were included in the model. As displayed in Table 5, fetal HbH disease was positively associated with an Apgar score < 7 at 1 min (OR, 3.37; 1.16–9.78) and an Apgar score < 7 at 5 min (OR, 4.50; 1.00–20.23). Gestational age was negatively correlated with an Apgar score < 7 at 1 min (OR, 0.63; 0.54–0.73) and an Apgar score < 7 at 5 min (OR, 0.65; 0.53–0.80). Therefore, fetal HbH disease and gestational age were two independent factors that influenced low Apgar scores.

**Table 5:** Binary logistic regression with backward elimination (Backward Likelihood Ratio), of factors associated with low Apgar score among thalassemic women.

Factors	Apgar score < 7 at 1 min			Apgar score < 7 at 5 min		
	OR	95% CI	*P*	OR	95% CI	*P*
Fetus with α-thalassemia trait (vs normal)	0.98	0.34–2.81	0.970	0.26	0.03–2.59	0.250
Fetus with HbH disease (vs normal)	3.37	1.16–9.78	0.025	4.50	1.00*–20.23	0.050^†^
Gestational age	0.63	0.54–0.73	< 0.001	0.65	0.53–0.80	< 0.001

*Lower limit of 95% confidence interval for odds ratio = 1.000024. ^†^*P* = 0.049996.

### Diagnostic value of single indicator and combination in predicting low Apgar scores

**Figure 2:**
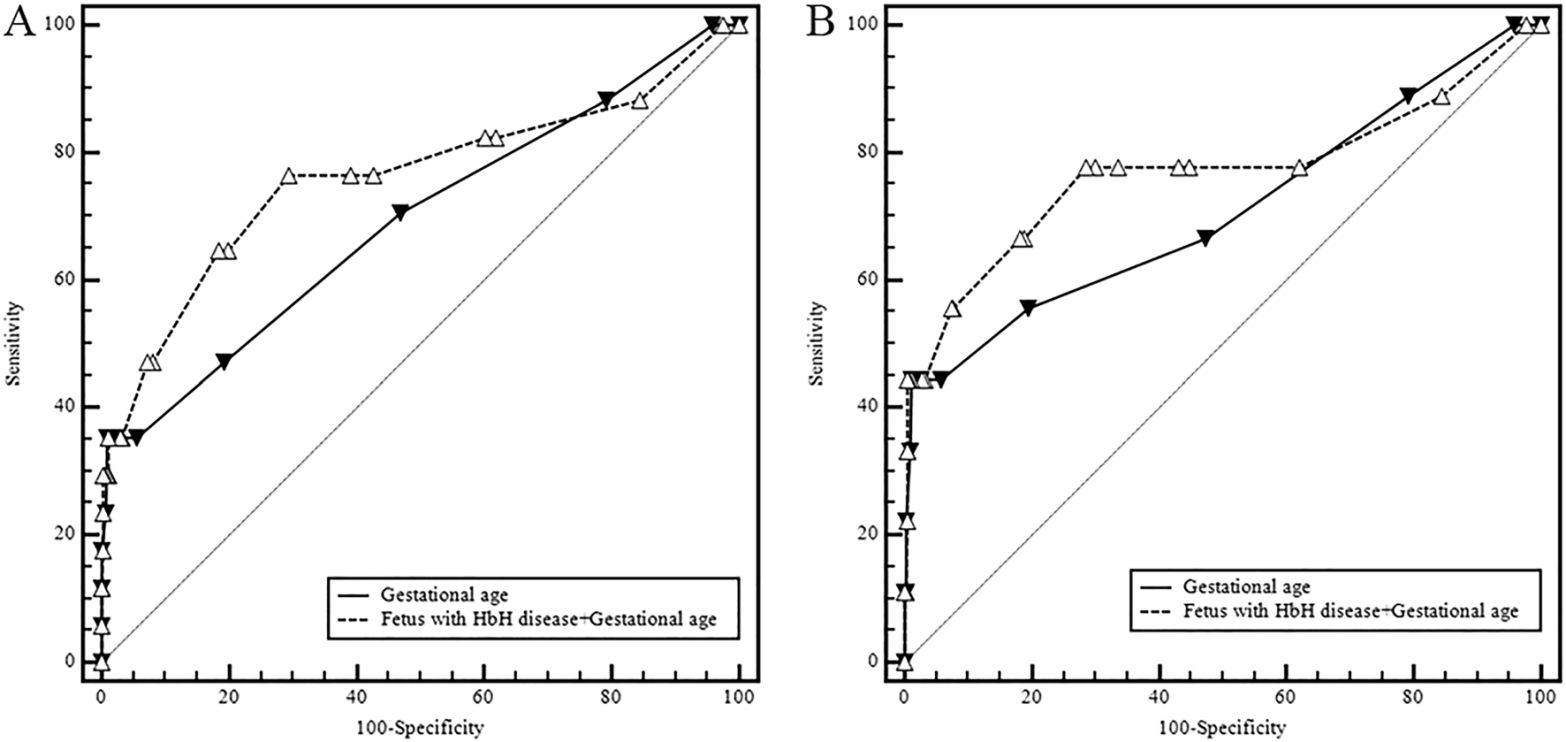
The receiver operating characteristics curves of gestational age alone and its combination with fetal HbH disease in predicting an Apgar score < 7 at 1 (**A**) or 5 (**B**) minutes among thalassemic women.

The results of ROC curve analyses showed that gestational age alone had a low accuracy in predicting an Apgar score < 7 at 1 min (AUC, 0.692; 0.655–0.727), with a sensitivity of 70.59% and specificity of 52.97%. Gestational age of 37 weeks had the best sensitivity (55.56%) and specificity (80.56%) in predicting an Apgar score < 7 at 5 min (AUC, 0.708; 0.672–0.743), where the *P* value was very close to the level of significance (Table 6; Fig. 2). When fetal HbH disease and gestational age were combined in the prediction of an Apgar score < 7 at 1 min and an Apgar score < 7 at 5 min, the AUC values were 0.760 (0.725–0.792) and 0.769 (0.735–0.801), respectively; the sensitivity values were 76.47% and 77.78%, respectively; and the specificity values were 70.47% and 71.45%, respectively. Combining fetal HbH disease and gestational age produced a medium accuracy in predicting an Apgar score < 7 at 1 or 5 min, although the AUC values were not significantly higher than those for gestational age alone (*P* = 0.076, *P* = 0.232, respectively).

**Table 6:** The sensitivity and specificity of gestational age alone and its combination with fetal HbH disease in the prediction of an Apgar score < 7 at 1 or 5 min among thalassemic women.

State variable	Test variable	Cutoff value	Sensitivity (%)	Specificity (%)	*P*	AUC	95% CI
Apgar score < 7 at 1 min	X1* (week)	38	70.59	52.97	0.013	0.692	0.655–0.727
	X2^†^	–	76.47	70.47	< 0.001	0.760	0.725–0.792
Apgar score < 7 at 5 min	X1* (week)	37	55.56	80.56	0.068	0.708	0.672–0.743
	X2^†^	–	77.78	71.45	0.020	0.769	0.735–0.801

AUC, area under the curve. *Gestational age. ^†^Fetus with HbH disease + Gestational age.

## Discussion

In this study, we demonstrated that fetal HbH disease was associated with increased odds for low Apgar scores, and that combining fetal HbH disease with gestational age could to some extent provide a predictor for low Apgar scores. In addition, thalassemic women whose fetuses were normal may have increased their risk for postpartum hemorrhage. However, α-thalassemia trait in fetuses was not associated with adverse pregnancy outcomes.

The influence of thalassemia in women on pregnancy outcome is well documented^13–17^, whereas the effect of thalassemia in the fetus on pregnancy outcome has been largely overlooked. Biochemical characterization has shown that Hb F (α2γ2) is formed by combining the α-globin and γ-globin gene products to constitute the major hemoglobin component of fetal red blood cells that predominates throughout most of gestation^1,2^; however, Hb A (α2β2), the other hemoglobin form that is comprised of α-globin and β-globin, accounts for the minority during the fetal period due to little expression of the β-gene^1,2^. In addition, the red blood cell indices such as Hb, MCV, MCH, and mean cell hemoglobin concentration (MCHC) are significantly different between normal fetuses and fetuses with α-thalassemia disease, but not between unaffected and affected fetuses with homozygous β-thalassemia disease^18,19^. For the above reasons, we assumed that fetuses afflicted with α-thalassemia instead of β-thalassemia might exert a significant impact on maternal and especially fetal outcomes of pregnancy. In this study, we were able to determine the influence of fetuses with α-thalassemia and its combination with maternal α-thalassemia on pregnancy outcomes, and this distinguishes ours from other studies^13,15,20^. Therefore, this study provided a more precise risk estimate of adverse pregnancy outcomes for both parents with α-thalassemia.

The percentage of individuals of Zhuang ethnicity was higher in thalassemic women compared with controls, implying that the Zhuang seem to show a genetic predisposition to α-thalassemia—probably due to a founder effect event initiated by genetic drift, isolated living conditions, and religious customs such as endogamous marriage^9^. Guangxi province is the region where most of the Zhuang live, and in this study, we found a higher percentage of residency in Guangxi province among thalassemic women relative to controls, which indirectly indicated a higher prevalence there. Thalassemic women had a higher number of prenatal visits > 10 because they needed to undergo more medical examinations to ensure that they were healthy enough to give birth to healthy babies. Additionally, higher incidences of anemia and nulliparity were found in thalassemic women, which was consistent with previous investigations^15,16,20^.

Some investigators who evaluated pregnancy outcome of women with HbH disease found a higher rate of low birth weight^13,21^. However, it was unclear whether fetuses with HbH disease had a direct effect on low birth weight. Interestingly, we observed no significant difference in the rate of low birth weight among the four groups, but the rate achieved statistical difference between non-thalassemic women and thalassemic women whose fetuses had HbH disease. Although the rate of low birth weight between the normal fetal group and the group of fetuses with HbH disease was not statistically significant (*P* = 0.060), it showed a tendency to increase. Thus, the relationship between fetal HbH disease and low birth weight requires further study.

Postpartum hemorrhage is the leading direct cause of maternal death worldwide, particularly in developing countries, including China^22,23^, and comparable rates of postpartum hemorrhage between thalassemic and non-thalassemic women have been found in previous studies^13,20^. Unlike the previous reports, however, we herein uncovered a higher rate of postpartum hemorrhage among thalassemic women whose fetuses were normal compared with controls. We postulated that the significant differences in red blood cell indices (e.g., Hb, MCV, and MCH) between mother and fetus—caused by maternal thalassemia—may be a potential cause for postpartum hemorrhage in the thalassemic women whose fetuses were normal. However, this hypothesis needs to be confirmed by further cohort studies.

Low Apgar scores have been associated with an increased risk of adverse pregnancy outcomes, mental retardation, and infant mortality^24–27^. In contrast with previous studies^13,21^, we uncovered a relationship between HbH disease and low Apgar scores, where fetuses with HbH disease were more likely to show lower Apgar scores compared with the normal fetal group. It was noteworthy that this trend was more obvious when non-thalassemic women were used as the reference. In the fetal period, a large excess of γ-chains instead of β-chains are produced and form γ_4_ tetramers (Hb Bart's) in fetuses with HbH disease^12^; however, the oxygen affinity of these homotetramers is too high to effectively deliver oxygen to tissues, increasing the odds of perinatal asphyxia. We hypothesized that the lower levels of Hb in maternal and particularly fetal blood (initiated by both showing α-thalassemia)^13,15,18,28^, may contribute to low Apgar scores.

Consistent with the previous study^29^, we herein found a significant negative association between gestational age and low Apgar scores among thalassemic women. However, we noted that predicting low Apgar scores based on gestational age alone was less accurate using a ROC curve. Intriguingly, when gestational age was combined with fetal HbH disease, we demonstrated a slightly better predictive value for low Apgar scores.

Several limitations to our study were also present. First, this study lacked data on fetal red blood cell indices (e.g., Hb, MCV, and MCH), because the technique for collecting fetal blood samples was invasive, and a large number of pregnant women showed a reduced willingness to support this work. This made it impossible for us to directly assess the impact of fetal anemia on pregnancy outcome. Second, our study—which was based on a multi-institutional database—was prone to possess investigational bias because it was difficult to standardize the measuring instruments used by different hospitals, although eligibility criteria were formulated to minimize this. Third, a small portion of the clinical data was absent or missing owing to retrospective collection, and may have affected our analytical results. Therefore, a prospective study will in the future contribute to confirming the results of our investigation.

## Conclusion

Our investigation conclusively demonstrated that the pregnancy outcomes of thalassemic women whose fetuses had the α-thalassemia trait were favorable. However, it was notable that a higher incidence of postpartum hemorrhage was found among thalassemic women with normal fetuses. More importantly, fetal HbH disease was associated with an increased risk for low Apgar scores. In addition, the combination of gestational age and fetal HbH disease presented a medium efficiency for predicting low Apgar scores. Owing to the deficiency in retrospective methodology, further prospective studies need to be performed to confirm the relationships between fetal α-thalassemia and specific pregnancy outcomes.

## Data Availability

The data used and analyzed during the current study are available from the corresponding author on reasonable request.
